# Effects of environmental change on agriculture, nutrition and health: A framework with a focus on fruits and vegetables

**DOI:** 10.12688/wellcomeopenres.11190.2

**Published:** 2017-10-31

**Authors:** Hanna L. Tuomisto, Pauline F.D. Scheelbeek, Zaid Chalabi, Rosemary Green, Richard D. Smith, Andy Haines, Alan D. Dangour

**Affiliations:** 1 Faculty of Epidemiology and Population Health, London School of Hygiene & Tropical Medicine, London, WC1E 7HT, UK; 2 Faculty of Public Health and Policy, London School of Hygiene & Tropical Medicine, London, WC1H 9SH, UK

**Keywords:** Environmental change, agriculture, nutrition, population health, climate change, food-systems, fruits, vegetables

## Abstract

Environmental changes are likely to affect agricultural production over the next  decades. The interactions between environmental change, agricultural yields and crop quality, and the critical pathways to future diets and health outcomes are largely undefined. There are currently no quantitative models to test the impact of multiple environmental changes on nutrition and health outcomes.

Using an interdisciplinary approach, we developed a framework to link the multiple interactions between environmental change, agricultural productivity and crop quality, population-level food availability, dietary intake and health outcomes, with a specific focus on fruits and vegetables. The main components of the framework consist of: i) socio-economic and societal factors, ii) environmental change stressors, iii) interventions and policies, iv) food system activities, v) food and nutrition security, and vi) health and well-being outcomes.

The framework, based on currently available evidence, provides an overview of the multidimensional and complex interactions with feedback between environmental change, production of fruits and vegetables, diets and health, and forms the analytical basis for future modelling and scenario testing.

## 1. Introduction

In the next decades, the world population will continue to be confronted with environmental changes that pose increasing challenges to our food systems, health and well-being. These changes – such as climate change, increased ground-level ozone, changes in water availability, carbon dioxide fertilisation, soil degradation, deforestation and land use change – can directly and substantially influence agricultural production. In addition, variability in abundance and spread of pests, pathogens and pollinators – which are also related to environmental change – could form an additional, indirect impact on agriculture. Without successful and widespread implementation of adaptation and mitigation strategies aiming to overcome and/or reverse these environmental changes and their consequences, global food security, health and well-being could be significantly affected ( IPCC, 2014).

The scale of impacts of environmental change on food systems and health will depend on a variety of environmental, behavioural and economic factors. Firstly, the magnitude of environmental change will depend on the current level and trends of different environmental stressors and the *mitigation* actions taken by both individual countries and the global society as a whole. For example, several countries are taking individual action to ban nicotinoid pesticides to protect insect pollinators, and the Paris agreement ( UN, 2015) has committed the global community to mitigating future climate change. Secondly, the effects of environmental change will depend on the *adaptation* mechanisms developed and adopted. This could include changing agricultural production methods and altering the types of crop grown in certain areas that are less sensitive to certain environmental stressors. Thirdly, markets play a key role in distributing food between production and consumption locations. Globalised agricultural systems may be better placed to respond to changes in environmental conditions for food production, whereas food systems in areas that are strongly dependent on local markets may be more vulnerable to environmental change. Fourthly, food prices have an influence on consumer behaviour – consumption of some foods is much more sensitive to price changes than other foods. Finally, the effect of changing food availability on nutrition and health is likely to differ between countries and population groups, due to both price responsiveness and differences in pre-existing dietary patterns. Therefore, predicting the impacts of environmental changes on diets and health requires a detailed understanding of the various interactions and feedback loops between numerous actors and processes, as well as information on environmental, social and economic contexts.

Past research has been largely one-directional and limited to single steps in the pathways linking environment, food and health, e.g. concentrating on the impacts of environmental change on crops or the impacts of different diets on health. Research related to the impacts of environmental change on food production has mainly focused on the effects of climate change on staple crops ( Challinor *et al.*, 2014; Knox *et al.*, 2012; Porter *et al*., 2014), whereas the impacts on other foods and impacts from other environmental stressors have been less studied.

A few studies have integrated environmental change, agriculture, markets, nutrition and health ( Myers *et al.*, 2017; Smith *et al.*, 2015; Springmann *et al.*, 2016a) focussing mostly on important staple crops and/or meat. These studies have provided better insight into the potential scale of the impact of environmental change on the food system but the nutritionally-important fruit and vegetable food-groups remain largely understudied. With their unique nutritional features, significance for public health and relatively low environmental footprint ( Clune *et al.*, 2017), fruits and vegetables have the potential to play a crucial role in healthy population diets of the future.

The association between low consumption of fruits and vegetables and risk of non-communicable diseases (NCDs) including cardiovascular diseases and certain types of cancer ( Forouzanfar *et al.*, 2016; Miller *et al.*, 2017) is well established. Furthermore, recent research has shown that even beyond the WHO recommendation of 400 grams a day, higher intake of fruits and vegetables continues to reduce risk of cardiovascular disease, cancer and all-cause mortality ( Aune *et al.*, 2017). The consumption of fruits and vegetables per person has been shown to be linked with socioeconomic status: low income countries have lower consumption per capita than high income countries ( Miller *et al.*, 2016a), and within countries consumption has been found to be lower in poor neighbourhoods than in wealthier ones ( Dubowitz *et al.*, 2008; Pessoa *et al.*, 2015). However, many fruit and vegetable crops prove to be relatively sensitive to environmental changes ( Backlund *et al.*, 2008) raising the prospect of reduced fruit and vegetable availability in the future with contingent public health concerns.

We focus in this paper specifically on fruits and vegetables due to their nutritional importance. The aim of this paper is to illustrate a set of pathways that connect environmental changes, production of fruits and vegetables, nutrition and health in a comprehensive framework. The framework provides a basis for the identification and detailed modelling of the key pathways that link environmental change – through agriculture and nutrition – with population health. Even though this paper focuses on fruits and vegetables, we acknowledge the importance of also considering staple crop and livestock production in a comprehensive analysis. Furthermore, the framework considers only pathways that impact health through nutrition, whereas direct health impacts of environmental changes (for example through air pollution, extreme weather events or infectious diseases) are not included in this paper.

## 2. Methods

The framework was constructed based on an extensive literature search, including both peer-reviewed and grey literature. First, the literature was searched for existing frameworks covering several parts of the environmental change, agriculture, nutrition and health nexus. The identified existing frameworks, such as Ingram (2011) and McMichael (2003), informed the selection of main components for the new framework and facilitated hypothesis formulation around impact pathways. Subsequently, evidence was gathered (preferably in the form of systematic reviews) to establish the main pathways linking environmental change (through agriculture) with nutrition and health. This exercise included consultations with experts working in the fields of environment, agriculture, trade, nutrition and health including those studying the temporal trends and impact of specific environmental stressors.

**Figure 1.**
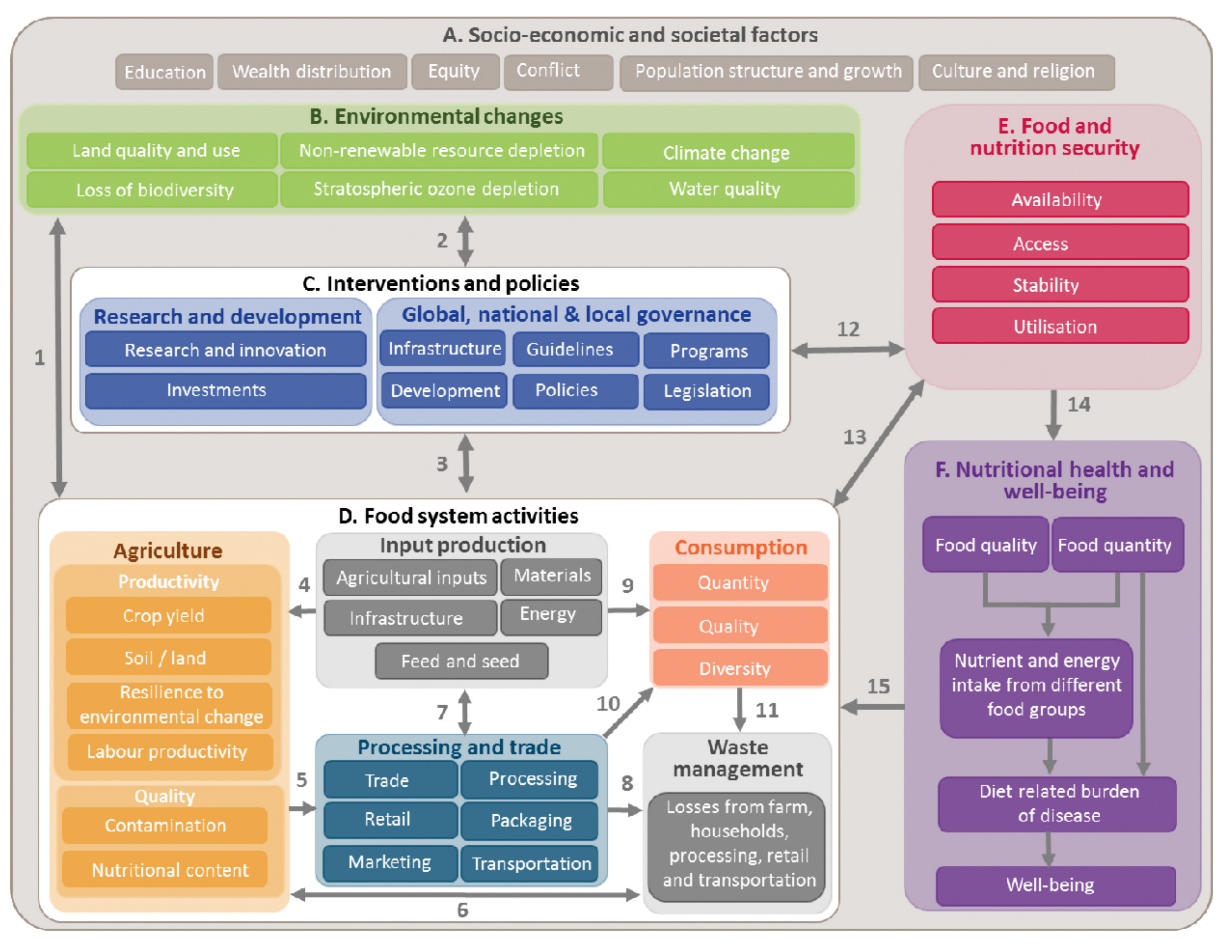
Overall framework connecting environmental change, agriculture, nutrition and health.

**Figure 2.**
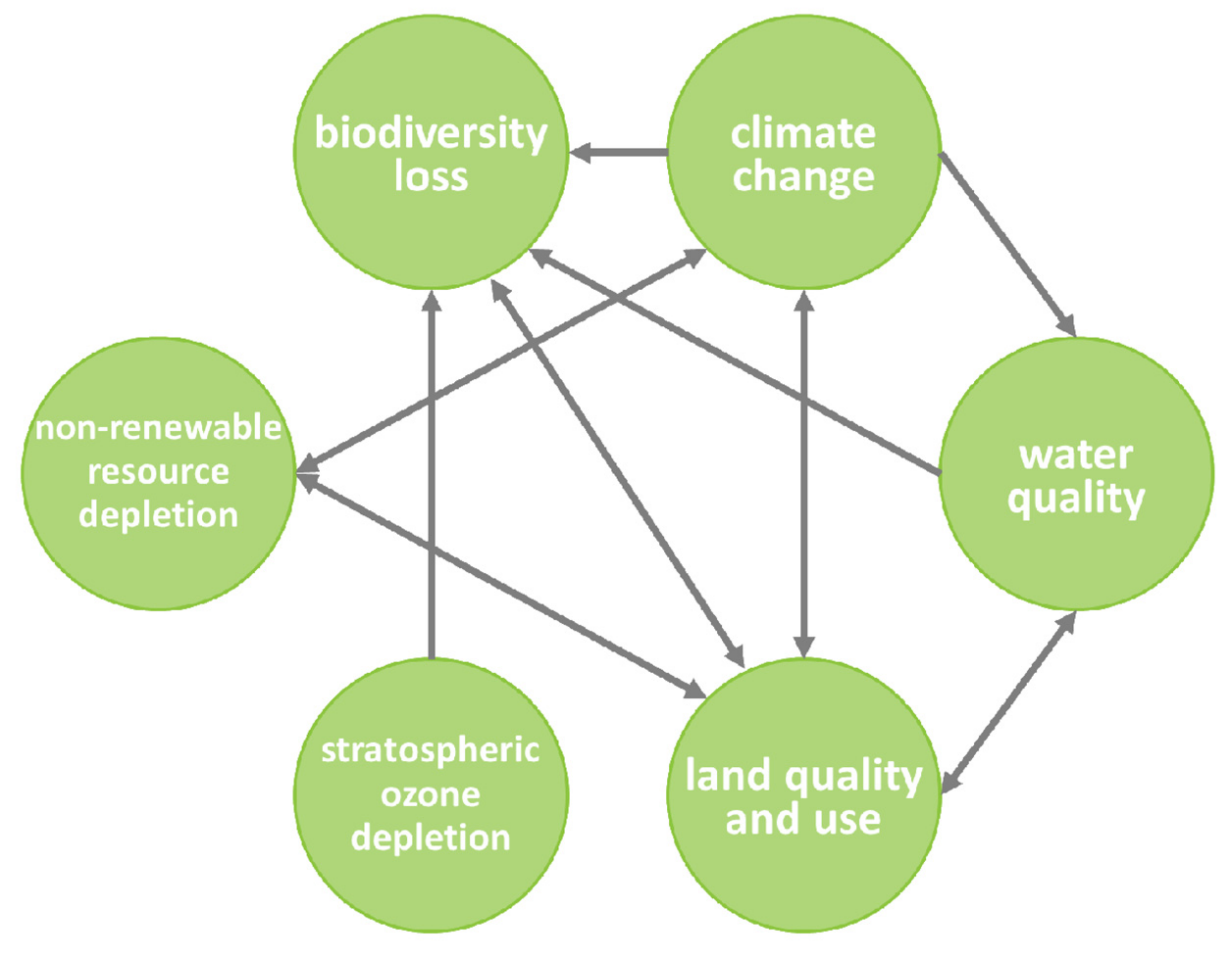
Links between environmental changes.

**Figure 3.**
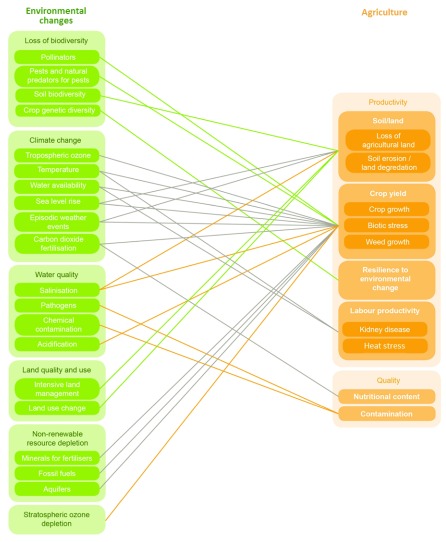
Pathways between environmental changes and agriculture.

The framework is graphically presented in three stages: i) a schematic overview of the links between environmental change, food systems, nutrition and health (Section 3, Figure 1); ii) illustration of the interactions between different environmental stressors (Section 4, Figure 2); and iii) the links between environmental stressors and production of fruits and vegetables (Section 4, Figure 3). The following section presents an overview of mechanisms through which the most important interactions between environmental change and production of fruits and vegetables operate (Section 4). The potential consequences of environmental change on food security (through changes in the availability of fruits and vegetables), nutrition and health outcomes are discussed in Section 5. The feedback loops from dietary choices to agricultural production and the impacts of agriculture on environmental change are discussed in Section 6 and the adaptation and mitigation strategies in Section 7. It was outside of the scope of this article to provide a systematic review of each interaction in the framework, neither was it possible to quantify and rank each individual stressor in terms of the strength of the evidence. We intend, however, to contribute to this evidence base through our future work.

## 3. Overall framework

Within the overall framework ( Figure 1), we refer to the boxes and the arrows in the figure with the symbols ■ and ▲, respectively, followed by a corresponding letter or number) six main components are distinguished to map the interactions between environmental change, agriculture, and nutrition: i) socio-economic and societal factors (■ A); ii) environmental changes (■ B); iii) interventions and policies (■ C); iv) food system activities (■ D); v) food and nutrition security (■ E); and vi) nutritional health and well-being (■ F) ( Figure 1). The socio-economic factors, such as culture, religion, wealth distribution and population structure provide the context for environmental change, interventions and policies, food system activities, level of food and nutrition security and nutrition related health and well-being. The environmental changes include stressors that directly affect food systems (▲1, *Section 4*). The interventions component includes research and innovation, technological development and government policies that provide the boundaries, opportunities and restrictions to the interactions between environmental changes, food system activities, food and nutrition security, health and well-being (▲2, 3, 12). The food system activities component covers the interlinked food system functions, including production of inputs and infrastructure, agricultural processes, food processing, trade, consumption and waste management (▲4–11). In the framework, food and nutrition security are identified as a fifth component group, which are important determinants of the burden of disease and well-being. The framework presents a static conceptualisation of the interactions, although we recognise that the interactions are dynamic and operate over different time scales. For example, changes in food prices can have an immediate impact on food consumption, whereas the impacts of some environmental changes on health outcomes may be seen only after a few decades.

## 4. Impacts of environmental change on production of fruits and vegetables

### 4.1. Climate change

Climate change has been predicted to impact agricultural production through multiple direct and indirect pathways ( Porter *et al*., 2014; Smith *et al*., 2014). Changes in temperature and water availability combined with increased variation in weather conditions and more frequent episodic weather events will have a direct impact on crop yields ( Lobell & Gourdji, 2012). Increased temperature results in faster crop growth, and therefore, shorter cropping seasons and lower yields. Temperature also impacts on photosynthesis rates and respiration. C4 crops (maize, sorghum, sugarcane, etc.) have higher optimum temperature for photosynthesis than C3 crops (cereals and most vegetables and fruits).

Climate change can have also some positive impacts as on crop production as increased carbon dioxide concentrations in the atmosphere can boost photosynthesis of C3 crops and water use efficiency in both C3 and C4 crops, and improve crop growth ( Long *et al.*, 2006). At the same time, however, this can lead to a reduction in protein, vitamin and mineral concentrations in the edible part of the crop, possibly due to reduced canopy transpiration or changes in metabolite or enzyme concentration ( McGrath & Lobell, 2013). This phenomenon was studied by Myers and colleagues who modelled the impact of CO _2_ on staple and legume crops and found that the impact of CO _2_ was very different for C3 plants compared to C4 plants ( Myers *et al.*, 2014; Myers *et al.*, 2015). Nearly all fruits and vegetables in the human diet are C3 crops and hence are likely to be relatively vulnerable to these climatic changes. While research on drought and heat resistant staple crops has taken off greatly in the last decades, adaptive capacities in fruits and vegetables are less studied.

Besides the direct effects, increased temperatures may indirectly affect fruit and vegetables yields due to decreased labour productivity of farmers, affecting agricultural productivity ( Kjellstrom *et al.*, 2016). Many fruit and vegetable crops require high labour inputs, especially for planting and harvesting and hence climate change induced heat stress may disproportionately affect this sector.

Climate change affects many other environmental drivers, both directly and indirectly ( Figure 2). For example, rising temperatures increase tropospheric (i.e. ground-level) ozone formation, and increased ozone levels cause oxidative stress for plants, which reduces photosynthesis and plant growth ( Ainsworth *et al.*, 2012). Furthermore, climate change has impacts on animal species, and a decrease of plant pollinator populations, for example, could have multiple impacts on agricultural production ( Pacifici *et al.*, 2015) (see Section 4.6). Climate change is also likely to increase crop losses and damages due to pests, pathogens, fungi and weeds ( Flood, 2010). It has been estimated that hundreds of pests and pathogens have moved towards poles on average by 2.7 km yr ^-1^ between 1960 and 2012 ( Bebber *et al*., 2013).

### 4.2. Historical ozone depletion & current ozone layer recovery

The stratospheric ozone layer, protecting the earth from solar ultraviolet (UV) radiation, has been depleting over the past decades due to anthropogenic emissions of chlorofluorocarbon and nitrous oxides, although the recent evidence indicates healing of the ozone layer due to reduced cholofluorocarbon emissions ( Solomon *et al*., 2016). However, in Antarctica, ozone depletion continues to occur each year, whereas the Arctic ozone shows high year-to-year variability ( Andrady *et al.*, 2015).

Many factors such as cloud cover, altitude, ground reflectance and atmospheric path length, impact on the level of UV-B reaching plants. Due to the natural variations of those factors, the effect of stabilization of the ozone layer is not yet detected in the measurements of UV-B radiation.

UV-B radiation has been found to damage DNA, RNA, proteins and membranes of plants and to impair photosynthesis ( Björn *et al.*, 1999; Caldwell *et al.*, 2007). A meta-analysis of the effect of increases in UV-B on yields found that herbaceous plants including most vegetables (e.g. beans, tomatoes, spinach, radish, carrots, cucumber and gourd) and many fruits (such as strawberries and sea-buckthorn) showed a more significant decrease in yield due to the UV-B exposure than woody plants ( Li *et al.*, 2010).

### 4.3. Water quality

The quality of irrigation water has a direct impact on crop quality and quantity. In the past decades, several trends in water quality – with a strong link to environmental change – have put increasing pressure on the agricultural sector, and it is expected that these trends will continue in the future ( Turral *et al.*, 2011).

Salinization is major threat to irrigation water quality. Salt tolerance levels vary greatly from crop to crop. Predominantly, salinization decreases yields, but the impact on crop quality is mixed ( Hoffman *et al.*, 1989). Many vegetable crops are negatively affected and salinity can substantially reduce their market value. However, in some crops, such as carrots and asparagus, salinity can increase sugar content, whilst in tomato and melon it can increase soluble solids. Generally, however, salinity-induced decreases in yield outweigh any beneficial effects ( Hoffman, 2010).

Climate change may exacerbate salinity problems which in turn impact health through drinking water and diet ( Khan *et al.*, 2014; Scheelbeek *et al*., 2017). In several low-lying coastal areas, the increased frequency of tropical cyclones and inundations can have a serious impact on the sodium (and other salts) content of soils as well as ground- and surface-water. In climate-vulnerable coastal areas, such as Bangladesh, an additional problem arises when farmers move away from saline irrigation sources and obtain water from deeper groundwater layers; high arsenic concentrations have been measured in these groundwater sources. Arsenic can remain on the crop’s surface after harvesting and could form a serious health threat to its consumers ( Das *et al.*, 2004; Su *et al.*, 2014). Further inland, changing precipitation patterns and drought can cause significant increase in sodium concentrations in freshwater bodies, affecting irrigation and drinking water quality ( Jeppesen *et al.*, 2015).

Contaminated irrigation water affects crop quantity and quality significantly. More than 10% of the global population consumes foods that are irrigated with untreated wastewater or faecal contaminated surface water, and most of those people live in low-income countries with arid and semi-arid climates ( WHO, 2006). Increasing water scarcity, expanding populations and recognition of the fertilisation value of wastewater are the main drivers for the increasing use contaminated water for irrigation. The use of pathogen (e.g. Salmonella spp., norovirus, E. coli, Clostridium perfringens and Cambylobacter spp.) contaminated urban wastewater for irrigation and post-harvest processes has been linked to food-borne disease outbreaks ( Antwi-Agyei *et al*., 2015; WHO, 2015). This is particularly a problem with fruits and vegetables that are often eaten without cooking.

Problems also occur if high concentrations of certain toxic ions in irrigation water - such as chloride, sodium and boron - are taken up by the plant and accumulate to concentrations that can cause damage in the crop and reduce yields ( Bañón *et al.*, 2011). Both agricultural and industrial factors play an important role in toxin concentrations in water, including chemical wastewater being released in watersheds used for agriculture and/or pumping up irrigation water, as well as farm-disposal of agrochemicals. Most irrigation water sources contain concentrations of elements below toxicity thresholds; however, boron tolerance of most vegetable crops is relatively low and even quite low boron concentrations could damage crops ( Hoffman, 2010). The magnitude of damage varies per crop; permanent perennial-type crops are believed to be most sensitive to irrigation water toxicity ( WHO, 2006).

A third important water quality threat is the occurrence of excessive nutrients in irrigation water, notably nitrogen. This is often the result of (over)fertilisation of agricultural land, whereby excess fertilisers end up in water sources used for irrigation and may damage marine ecosystems. In susceptible crops - such as apricot, citrus and avocado - high nitrogen concentrations trigger excessive vegetative growth and delay of maturing. In leafy vegetables, this causes a decrease in harvestable product and could negatively affect fruit quality parameters, such as sugar content ( Ayers & Westcot, 1985). It could also cause crops to grow taller and hence to be more vulnerable to lodging (bending over of stems) in extreme weather events, such as tropical storms.

### 4.4. Non-renewable resource depletion

Non-renewable resource depletion includes reduced availability of minerals used for fossil fuels, fertilisers or infrastructure, and depletion of aquifers that can be used for irrigation water. The reduced availability of these resources can have an impact on crop production, unless alternative technologies are adopted (e.g. use of renewable energy sources or organic fertilisers).

For example, it has been estimated that the current economically exploitable phosphate reserves will be depleted in approximately 50–100 years ( Cordell *et al.*, 2009). Therefore, options to recycle nutrients back to the fields from bio-waste and sewage sludge may become more financially attractive. Similarly, industrial agriculture relies heavily on the use of fossil fuels for producing nitrogen fertilisers, running farm machinery and other uses. The depletion of fossil fuel reserves or the inability to exploit them because of climate change imperatives may pose a threat for agricultural production unless renewable energy sources can be significantly scaled up. However, this will be more of a problem in industrial farming systems than in subsistence farming that relies mainly on manual labour.

Finally, the depletion of water resources can have negative impacts on agricultural production, especially in areas where aquifers provide an important source of irrigation water. The depletion of aquifers is linked to changes in precipitation levels, exhaustion of rivers and increased use of water. Climate model simulations project precipitation increases in high latitudes and parts of the tropics, and decreases in some tropical and lower mid-latitude regions ( Bates *et al.*, 2008). Poor rural farmers in the arid and semi-arid tropics and Asian and African mega-deltas are likely to be the most vulnerable to these changes in water availability. Furthermore, international food trade contributes to the decline of aquifers in the producing countries ( Dalin *et al.*, 2017). Most of the irrigation water globally is used for staple crops (mainly for wheat) and less than 10% of all irrigation water is used for fruits and vegetables, which is in line with the percentage of land used for fruits and vegetables ( FAOSTAT, 2017).

### 4.5. Land use

Agricultural land is a limited natural resource. It is estimated that nearly a third of global arable land has been lost due to soil erosion and pollution during the past 40 years ( Cameron *et al.*, 2015). Other reasons for loss of agricultural land include urbanisation, sea level rise, and renewable energy production (e.g. solar panels on agricultural land), as well as land requirements for bio-fuels and other non-food crops. At the same time, forests have been converted to agricultural land, mainly driven by increased consumption of meat and need of land for feed production. Therefore, the percentage of agricultural area of the total global land area has been relatively stable during the past decades. However, deforestation contribute to the acceleration of many environmental changes, such as climate change and loss of biodiversity, and therefore, can have negative indirect impacts on food security, e.g. through loss of wild foods (Section 6). 

Soil degradation typically refers to multiple processes, such as erosion, desertification, salinization, compaction and encroachment of invasive species ( Gibbs & Salmon, 2015). Soil organic matter plays a vital role in maintaining the long-term productivity of soils. The increased use of industrial farming practices, such as mono-cropping, minimal use of organic fertilisers and removal of crop residues from fields, is one of the main reasons for decline in soil organic matter contents.

Acidification of soils is caused by acid rains or use of synthetic nitrogen fertilisers in some conditions. Acid rains generally result from the reaction of water molecules and sulphur dioxide or nitrogen oxide in the atmosphere, which mainly originate from anthropogenic sources, such as energy generation and industrial processes ( Klimont *et al.*, 2013). Soil acidification can alter nutrient availability, and has generally negative impact on plant growth, except in alkaline soils some acidification can be beneficial ( Lee *et al.*, 1981). Application of lime and balanced fertilisers help to mitigate crop losses caused by acidification ( Mason *et al.*, 1994).

Phytotoxicity means the toxic effect on plants caused by compounds such as trace metals, allelochemicals, pesticides, phytotoxins or salinity. Contamination of soil with toxic metals, such as cadmium and high concentrations of aluminium, has negative impacts both on crop yields and human health ( Khan *et al.*, 2015). Metals cause oxidative stress for plants, which reduces biomass accumulation.

### 4.6. Biodiversity loss

In some cases, losses of biodiversity can have direct impacts on food availability in areas where wild food, including wild fruits and vegetables, comprise a substantial proportion of diets. Field-grown crops and livestock are also heavily dependent on multiple ecosystem services, such as pollination, natural predation of pests and services provided by soil macro- and micro-organisms.

During the past decade, the numbers of pollinators have declined, due to combined stress from parasites, pesticides and habitat loss ( Goulson *et al.*, 2015). As many fruit and vegetable species rely on pollinators, a complete loss of pollinators has been predicted to reduce global fruit supply by 23%, vegetables by 16% and nuts and seeds by 22% with major adverse effects on health ( Smith *et al.*, 2015).

Ecosystem functions are complex and it is currently not possible to model the required level of biodiversity needed for sustaining agricultural production. Therefore, maintaining a high level of biodiversity is regarded as a precautionary mechanism that increases the resilience of agro-ecosystems to environmental changes ( Koohafkan *et al.*, 2012; Lin, 2011). Farming practices that reduce vulnerability to environmental change include diversification of agro-ecosystems, high genetic diversity of crops, integration of livestock and crop production, management of soil organic matter and water conservation. Crop diversification reduces pest, disease and weed outbreaks, and increases resilience towards greater climate variability and extreme events. In low income settings, farms with a high level of biodiversity have been found to be more resilient to climate disasters, such as hurricanes and droughts ( Altieri *et al.*, 2015). Smallholder farmers in tropical regions are particularly vulnerable to climate variability, including erratic rainfall, and as a coping mechanism they rely on agricultural biodiversity, such as planting a high diversity of crops each year, including many varieties of the same crop, using drought tolerant crop varieties, changing the locations of crops and planting trees to provide shade and to maintain humidity ( Meldrum *et al.*, 2013).

## 5. Impact of drivers, influencers and activities on food security and health outcomes

### 5.1. Links between agriculture and food security: From subsistence farming to international trade

The most direct link between agriculture and food security occurs in subsistence farming communities and involves the production and quality of crops and their impact on the availability of nutritious food to producing households. Most people living in the rural areas in low income countries, especially in sub-Saharan Africa, are dependent from subsistence farming, and 72% of all farms in the world are under 1 hectare ( FAO, 2014; Herrero *et al.*, 2017).

Considering the predominantly negative influences of environmental stressors on both fruit and vegetable yield and quality (see previous sections), populations heavily reliant on subsistence farming appear likely to have food insecurity in the future ( Morton, 2007; Shrestha & Nepal, 2016; Tibesigwa *et al.*, 2015). The extent of these influences on their nutrition and health depends on the farmers’ ability to adapt to these environmental changes ( Shisanya & Mafongoya, 2016). Many subsistence farmers are particularly vulnerable due to a high dependence on rain-fed agriculture and limited adaptation strategies: rain-fed agriculture accounts for approximately 95% of farmed land in sub-Saharan Africa and 90% in Latin America ( Wani *et al.*, 2009). Moreover, in contexts where agricultural surpluses are sold at the local market as critical sources of cash, reduced yields will likely decrease household incomes.

In larger and more complex trade systems – ranging from farmers producing for the local markets to agribusinesses and international trade – a more complex interplay of mechanisms determine the impact of suboptimal yields on food security, including market mechanisms and food choices ( Figure 1, ■ D), possible technological or political interventions ( Figure 1, ■ C) and the influence of social factors ( Figure 1, ■ A).

Compromised production – and therewith reduced availability – of a locally important vegetable could, for example, push up local or regional prices, and make the specific vegetable unaffordable for the less affluent ( Brown *et al.*, 2012). Households’ purchasing power and preference will determine their substitution strategy, e.g. buying another cheaper vegetable if available, buying more staples, or not substituting the “missing” vegetable. The price elasticities of fruits and vegetables tend to be higher than those for cereals, which means that consumers reduce their demand more in response to an increase in price ( Cornelsen *et al.*, 2015). The household substitution strategy used will partly determine the scale of health impacts ( UNSCS, 2010).

Forced switches to alternative crops could also have far reaching consequences for farmers, in case the switches become permanent (i.e. consumers start preferring the “new” vegetable above the “conventional” one), as sometimes experienced after temporary food aid programmes ( Barrett, 2006). This applies especially to small farmers that might lack the financial resources to shift to another (more commercial) crop as a response to the changed commodity prices, even if this would be much more profitable ( García-Germán *et al.*, 2013). Higher prices may push subsistence farmers to sell more and consume less of their own yields, which could also have an impact on their food security ( Anríquez *et al.*, 2013; Zezza *et al.*, 2008). Nonetheless, it has been argued that higher food prices will generally affect food security of net consumer countries more than net producer countries ( ODI, 2008), and nutritional health, especially among children under 5 years of age ( Figure 1, ▲13, 14). In larger markets with more producers integrated across diverse environments, the abundance of competitors offering the same vegetable crop may stabilise the commodity prices, and may therefore directly affect the financial security of farmers that experienced compromised yields of that specific vegetable.

Crop quality, including nutritional content, may affect dietary micronutrient supplies of consumers and subsistence farmers. Especially in areas where nutritional needs are only marginally met or where there is a widespread marginal nutrient deficiency, slight changes in vitamin and mineral concentration in crops – even without any actual change in diet – could be crucial for food and nutrition security. Fruits and vegetables are therefore particularly important as they provide a rich source of essential micronutrients that are present in much lower concentrations in other food groups.

### 5.2. Links between food security, consumption, health and well-being

There is a substantial evidence base on the impact of food security on population diets. Furthermore, the links between diets, health and well-being are the most well-researched parts of the framework ( Figure 1,▲14). Non-optimal diets are estimated to account for ~10% of the global burden of disease ( Forouzanfar *et al.*, 2016).

There are two main pathways leading from nutrition to population health: non-optimal *quantity* of food intake (under- and over-nutrition) and non-optimal *quality* of food intake (nutrient deficiencies due to poor dietary composition, toxins, pathogens, etc.). In terms of the former pathway, overweight and obesity increases the risk of various NCDs, including diabetes, certain cancers and cardiovascular disease, whilst undernutrition can lead to several deficiencies, affecting, for example, child growth and development and immune system function ( Figure 1, ■ F).

As well as contributing to daily dietary energy requirements, fruits and vegetables play a key role in the second pathway, linking sub-optimal quality of food intake and poor health. For many populations around the world, fruits and vegetables provide several essential vitamins, minerals and amino acids usually found in limited amounts in other components of the diet, particularly where consumption of animal-source foods is low. Low fruit and vegetable intake is associated with increased risk of vitamin deficiencies, all-cause mortality, coronary heart disease, strokes, and several types of cancer ( Forouzanfar *et al.*, 2016; Miller *et al.*, 2017; Wang *et al.*, 2014).

To further explore the importance of the pathway between fruit and vegetable consumption and health, full dietary composition (i.e. consumption besides fruits and vegetables) should be considered, as well as the drivers for food choices. Low fruit and vegetable intake can in some situations be the direct results of food insecurity (i.e. limited access, affordability of stability of fruits and vegetables), whilst in other situations it reflects the population’s preferences to consume foods high in sugar, salt and saturated fats instead of fruits and vegetables.

Where clinical health outcomes are difficult to measure, anthropometric indicators, such as height-for-age, weight-for-height and biomarkers, including cholesterol level, blood pressure and blood glucose, can be used for modelling the health implications of a diet.

## 6. Feedback loops from dietary choices and agriculture to environmental change

The framework highlights that – in addition to the described “environment – food system – health” pathway – there are several feedback loops linking dietary choices and nutrition back to agricultural strategies ( Figure 1, ▲15) and environmental change ( Figure 1, ▲1).

A remarkable example of these feedback loops is based on the rapid global shift towards a more “Western” diet, which is driven by urbanisation, economic growth and changes in technology and culture ( Popkin, 2006; Tilman & Clark, 2014). Western diets are characterised by greater consumption of animal source and highly processed foods often in parallel with a reduction of the consumption of vegetables and pulses. To meet the growing demand in animal source products, livestock and dairy farming has increased enormously ( FAO, 2015), contributing directly to increased greenhouse gas emissions, eutrophication (the enrichment of an ecosystem with nutrients), and loss of biodiversity due to intensification of agriculture and conversion of forests and natural habitats to agricultural land ( Gerber *et al.*, 2013). Currently, livestock production occupies approximately 80% of global agricultural land (including arable and grassland), whereas only a few percent of the land is used for fruits and vegetables ( FAO, 2017).

Agriculture is also one of the main contributors to climate change, accounting for ~25% of global anthropogenic emissions ( Vermeulen *et al.*, 2012), while livestock production alone has been estimated to account for 14.5% of global greenhouse gas emissions ( Gerber *et al.*, 2013). It has been estimated that the consumption of fruits and vegetables accounts for only 7% of all food related GHG emissions globally ( Springmann *et al*., 2016b). Generally, fruits and vegetables have a lower carbon footprint compared to livestock products and grains when measured per unit of product weight, although this is not necessarily the case when measured per unit of energy content, especially if the fruits and vegetables are processed ( Drewnowski *et al.*, 2015).

Agriculture is estimated to account for ~70% of global water withdrawals ( Mekonnen & Hoekstra, 2010). The water footprint of fruits and vegetables is relatively low compared to cereals and oil crops when measured per unit of product, but higher when measured per unit of energy. However, the variation between different fruits is high - ranging from 235 m ^3^ water per tonne of watermelon to 3350 m ^3^ water per tonne of figs ( Mekonnen & Hoekstra, 2010).

Particularly in developed countries, agriculture is the main contributor to eutrophication of waterways, due to nitrogen and phosphorus leached from fields ( Withers *et al.*, 2014). Eutrophication disturbs the natural balance of the ecosystem by favouring certain species and causing harm to others, e.g. in aquatic ecosystems the nutrient inputs increase the growth of algae and plants, and the decay of the biomass leads to oxygen depletion, causing death of fish and other aquatic animals. The eutrophication potential of fruit and vegetable production is generally higher than that of cereals ( Xue & Landis, 2010), due to the relatively high nutrient inputs required for production of fruits and vegetables.

Agricultural emissions, such as ammonia, toxic organic compounds, pesticides and particulates, have an impact on air quality, which has direct implications for human health. Agriculture accounts for ~30% of all acidifying emissions and 90% of ammonia emissions in Western Europe ( Erisman *et al.*, 2008). Ammonia emissions are mainly produced from manure management and use of nitrogen fertilisers. The contribution of agriculture to particulate matter emissions in Europe has been estimated to be ~20% ( Erisman *et al.*, 2008). Particulate matter emissions from agriculture originate from field operations such as ploughing, tillage and harvesting, and from livestock bedding materials and manure.

Industrialisation of agriculture has also contributed to the losses in biodiversity due to simplification of agroecosystems, reduced number of crops and crop varieties grown, use of chemical fertilisers and pesticides, intensification of agriculture, increase in field size and clearance of natural forests for agricultural land. The increased demand for agricultural products is causing a pressure for converting forests to agricultural land, especially in tropical regions ( Laurance *et al.*, 2014). Extensive farming systems, such as organic farming systems, generally have higher on-farm biodiversity compared to intensive farming ( Bengtsson *et al.*, 2005; Tuomisto *et al.*, 2012a). However, many studies have questioned whether land sparing, i.e. using intensive farming systems and leaving land out from agriculture for biodiversity conservation would lead to higher total biodiversity benefits compared to land sharing ( Phalan *et al.*, 2011; Tscharntke *et al.* (2012); Tuomisto *et al.*, 2012b points out that there is a clear difference between the type of biodiversity that land sparing and land sharing approaches support. The land sparing concept can under value functional agrobiodiversity that helps to increase the resilience of the farming systems to environmental changes.

## 7. Adaptation and mitigation options

There are many possibilities for farmers and societies to adapt to and mitigate environmental changes ( FAO, 2010; FAO, 2012). These practices can happen at various levels and range from minor changes to major system level changes. The agriculture and food production industries can implement adaptation practices that ensure increased high-quality food production with lower environmental burdens. However, as increasing food production does not guarantee that food would be distributed equally, additional policies will be required to improve the availability and access to healthy and nutritious foods to everybody. 

Farmers have possibilities to adapt to environmental changes by altering farm management practices, such as changing crop varieties, planting times, irrigation practices and residue management, or by implementing major systemic changes, such as switches to different crop species and changes in farming systems or even relocation of agriculture to new areas ( Challinor *et al.*, 2014). Many farming practices that increase the climate resilience of agriculture also help to mitigate GHG emissions ( Altieri & Nicholls, 2017).

Agriculture can also benefit from technological innovations, such as biotechnology and precision farming. Novel plant breeding technologies can provide crop varieties that are more suitable to new environmental conditions, e.g. drought resistant crops ( Hu & Xiong, 2014), or have higher concentrations and bioavailability of micronutrients ( Bhullar & Gruissem, 2013). Precision farming technologies apply geographical information systems, remote sensing and GPS for identifying variations in fields, and therefore help farmers to target the use of fertilisers and pesticides where they are needed the most. Small unmanned aerial systems are increasingly used for field imaging to find the problem areas at an early stage ( Zhang & Kovacs, 2012). The use of robots in agriculture is increasing, especially for activities that are currently often carried out manually, e.g. weed control, fertilisation and harvesting of fruits and vegetables ( Bogue & Bogue, 2016). The replacement of human labour by robots can be extremely beneficial as an adaptation to climate change, especially in areas where high daytime temperatures will make working on the fields impossible.

Novel technologies can also provide solutions to more systemic changes. Indoor farming and cellular agriculture enable food production without direct exposure to environmental stressors. Indoor farming in vertical systems (e.g. tall buildings) reduces land requirements and transportation needs, as production can take place closer to cities. The need for artificial lighting in many indoor farming systems is energy consuming ( Cheng, 2014), but developments in LED light technology may improve the energy efficiency of those systems in the future ( Darko *et al.*, 2014).

Cellular agriculture or the production of agricultural products by using cell culturing technologies, has the potential to revolutionise food production. The products from cellular agriculture include both acellular and cellular products. Acellular products are produced by culturing yeast or bacteria that synthetize a protein (e.g. milk protein or egg albumin) that is used for the final product. Cellular products, such as cultured meat or leather, consist of living or once living cells ( Post, 2012). Cellular agriculture is not limited only to replacing animal source foods, but plant cells and algae can also be cultivated in bioreactors for food ( Räty, 2017). Most of these technologies are currently at the development stage, but commercial products are expected to appear in the supermarkets during the next few years. Some preliminary studies have estimated that products from cellular agriculture could have potential to reduce environmental impacts substantially compared to conventionally produced livestock products ( Mattick *et al.*, 2015; Tuomisto & de Mattos, 2011; Tuomisto *et al.*, 2016). Studies on the environmental impact of plant products produced through cellular agriculture are currently lacking.

Adaptation and mitigation mechanisms are required also in the post-farm/post-primary production stage. Extreme climatic and hydrological events can make transportation of food less reliable due to floods, heavy rains, landslides etc. Therefore, diversification of supply chains and increased local production may increase the resilience and stability of food supply chains ( Miller *et al.*, 2016b). This may require food industries and consumers to adopt purchasing strategies that take into account seasonality based on the local climate. However, relying solely on local production is not a secure strategy due to the risk of extreme climatic and hydrological events affecting the local area.

Consumers have also a key role to play as they have the power to influence in the sustainability of food system by their consumption behaviour and dietary choices. As discussed in section 5&6 the consumption choices regarding quantities of animal source foods have a major impact on the environmental burden of diets. Environmental changes may also require consumers to alter the consumption of fruits and vegetables, as the availability and prices of most popular products may change. Therefore, consumers might need to choose different fruits and vegetables at different seasons and get used to a wider variety of species. Purchasing locally produced commodities could also promote the expansion of local production.

## 8. Conclusions

The evidence-based framework presented in this paper provides an overview of the multidimensional and complex interactions with feedback between environmental change, the food system, nutrition and health, and forms an analytical basis for detailed investigation of these interactions. The novelty of the framework is in its focus on fruits and vegetables, and in the detailed presentation of the pathways between environmental stressors and plant production ( Figure 3). This paper emphasises the importance of considering multiple environmental stressors and their interactions instead of focusing only on a single stressor (e.g. climate change). The focus on fruits and vegetables highlights the need for more research on this nutritionally-important food group as the majority of research efforts to-date have been targeted on staple crops and animal source foods. 

The framework can be adapted for other food groups as well as for regional case studies. The inclusion of the livestock sector would require adding livestock specific pathways into the framework, such as changes in livestock diseases and changes in grassland quality and feed production. The current framework can be directly used for staple crops.

This paper has highlighted many environmental issues that can potentially have major nutrition and health consequences unless mitigation and adaptation practices are implemented. However, many of the major risks may be faced by farmers and poor consumers in developing countries whose adaptation possibilities are limited especially in the short term. Therefore, this framework helps to develop further research to estimate the potential nutrition and health consequences of environmental changes on different population groups, and the effectiveness of alternative mitigation and adaptation options with various timeframes.

Some other more specific potential applications of the framework include:

- Guiding our understanding of the complex interactions of environmental, social, political, agricultural, market-related food security, diet and health mechanisms within food systems. It could be used for teaching and training sessions, research priority settings, as well as advocacy purposes.
- Identifying research gaps, determining research directions and guiding proposal writing. Likewise, the information can be used by funders to specify calls for proposals.
- Use as a heuristic tool for future food system and multi-sectoral modelling. This will enable further quantification of the impacts of environmental change – through agriculture and food security – on population health, as well as the assessment of the effectiveness of adaptation mechanisms at different parts of the system. By using an open-source platform, further detail could be added to the framework – and shared with the research community – when more evidence will become available.
- For food system programmes and policy makers, the framework gives an overview of where in the food system there are barriers and opportunities for change. With the available evidence, it would be possible to identify crucial links and mechanisms, which can guide health and sustainability programmes, as well as food system policy formulation.
- Although the framework was written for environment, food system and health interactions, similar frameworks could potentially be constructed in other sectors. The key role and interactions that societal factors, policies and research play within the “core” system mechanisms, is something commonly observed in other sectors (e.g. urban planning). The framework provides an example of how these complex interactions can be captured.
