# Supplementary material for: Effects of environmental change on agriculture, nutrition and health: A framework with a focus on fruits and vegetables
Source: Wellcome Open Res. 2017 Oct 31;2:21. Originally published 2017 Mar 28. [Version 2] doi: 10.12688/wellcomeopenres.11190.2 (PMC5814744; doi:10.12688/wellcomeopenres.11190.2)
Supplement: Peer review history

# Peer review: Referee response for version 2

**DOI:** 10.21956/wellcomeopenres.13915.r30892

**Published:** 2018-02-15

**Authors:** John McDermott

**Affiliations:** 1 CGIAR Research Program on Agriculture for Nutrition and Health (A4NH), International Food Policy Research Institute (IFPRI), Washington, DC, USA

In this second review, I will restrict my comments to how the authors have responded to my initial review.

 As in most work on food systems, the challenge is to investigate complex and dynamic systems and then consider the implications of different actors and actions in the system. Actors and actions may operate at different scales (global, continental, national and sub-national), in different food commodities (plant or animal, cultivated or gathered) and at different stages in the food supply chain (production, logistics, processing, retail and meal preparation). I appreciate the authors efforts in revising this paper to more realistically focus the paper on fruits and vegetables while considering broader system variables. The title and content more accurately reflect that focus.

 I am also pleased that the authors have acknowledged and partially addressed the differences between food systems in low- and middle-income countries and rich countries. This is a fundamental issue. In low-income countries 40-80% of people are engaged in agriculture and the agricultural sector accounts for approximately 20-40% of national GDP. With national economic “development” and increasing incomes in countries, the percentage of people engaged in agriculture declines dramatically (<2% in most high-income countries) and most of the value addition in the food systems occurs beyond the farm in logistics, processing, retail and food preparation. Because agriculture is such a major sector in LMICs and has profound environmental implications, I do appreciate the acknowledgement by the authors of the current article’s limitations and the need for further research in the agricultural-environmental implications of food system transformation in LMICS.

 With the narrowing of the focus of the article, some of the other environmental concerns I raised are less critical. Relative to habitat change and biodiversity conservation, the expansion of fruit and vegetable production into forests is less important than for more extensive agriculture. The bigger habitat issue for fruits and vegetables will be around the environmental impact on natural wetlands, which are often major sites for fruit and vegetables production. Also, given the perishability of fruits and vegetables, they are often produced close to or in urban areas. In urban and peri-urban settings, more attention to spatial analysis is warranted, particularly relative to important consumer concerns such as microbial and chemical contamination.

I have read this submission. I believe that I have an appropriate level of expertise to confirm that it is of an acceptable scientific standard.

# Peer review: Referee response for version 2

**DOI:** 10.21956/wellcomeopenres.13915.r27449

**Published:** 2017-12-18

**Authors:** Marco Springmann

**Affiliations:** 1 Oxford Martin Programme on the Future of Food, Centre on Population Approaches for Non-Communicable Disease Prevention, Nuffield Department of Population Health, University of Oxford, Oxford, UK

In their revision, Hanna Tuomisto and colleagues have addressed my comments. The manuscript provides a good overview of the various linkages of the food system with environmental change, and I think it will make a useful addition to the literature. I approve the manuscript and recommend it for publication/indexing. There are just three small comments, the authors might want to consider before that.

 First, the in their abstract, it is stated that '[t]here are currently no quantitative models to test the impact of multiple environmental changes n nutrition and health outcomes." I find that a rather strong statement, in particular as several research groups are working on such models, and some existing ones could be interpreted as being in that realm. Thus, the statement is or might be out of date soon. In addition, the manuscript does not address this gap, because it focuses on developing a qualitative framework. I would therefore suggest to omit it.   

 Second, in section 5.2, it is stated that "[n]on-optimal diets are estimated to account for ~10% of the global burden of disease (Forouzanfar et al., 2016)." I find that statement a bit unclear. For example, if one looked at the percentage of attributable deaths that was due to dietary risks, then it was actually 11 million out of 31 million (~35%), and if one looked at attributable DALYs, the reported estimate was 241 million out of 997 million (~24%), both a bit higher than the 10% that was reported in the manuscript. The attributable disease burden that was due specifically to diets low and fruits and vegetables in 2013 were 17% and 11% for deaths and DALYs, respectively. I would suggest to clarify the statement in the manuscript.  

 Third, would it be possible to supply Figure 1 in a higher resolution? In the current version, it appears to be rather blurry.

I have read this submission. I believe that I have an appropriate level of expertise to confirm that it is of an acceptable scientific standard.

# Peer review: Referee response for version 1

**DOI:** 10.21956/wellcomeopenres.12073.r22178

**Published:** 2017-05-30

**Authors:** Marco Springmann

**Affiliations:** 1 Oxford Martin Programme on the Future of Food, Centre on Population Approaches for Non-Communicable Disease Prevention, Nuffield Department of Population Health, University of Oxford, Oxford, UK

In their article ‘Effects of environmental change on population nutrition and health: A comprehensive framework with a focus on fruits and vegetables,’ Hanna Tuomisto and colleagues aim to develop a framework that details the interactions between environmental change, diets, and health, with a particular focus on fruits and vegetables. Their article is a welcome review of the impacts of environmental change on agriculture and health, and I recommend it for indexing subject to addressing a few comments that I am detailing below.

 First, I think the motivation of the review could be strengthened. What sets it apart from other reviews, such as the IPCC’s, or maps of the food system? Related to that, the article does not contain any methods and discussion sections. This might be fine for a review/overview article, but if the stated aim is to develop a framework of interactions, then one would expect at least some detail on what the added value of that framework is, how it was constructed, and how it compares to other frameworks. From my reading of the article, it is a review of interactions between environmental change and mostly agriculture, with special emphasis on the implications for fruits and vegetables, and some discussion on health implications. It might therefore be advisable to describe it as such.

 That would also address some problems I have with the conclusions, which seem to be a little bit of an overstretch to me. For most of the points raised, what would actually be required is some information on the relative importance of each factor. For advocacy or funding purposes, for example, one would want to know how significant a particular aspect is to gauge whether focussing on it would be worth the investment. The review, I think, nicely catalogues the various interactions between environmental change and agriculture, but it does not contain any interpretation of the information that is presented, or a discussion on what to do with it.

 For some of the aspects that are discussed I found myself going back to related IPCC chapters, in particular those on Agriculture, Forestry and Other Land Use (AR5, WG3, Chapter 11), Food Security and Food Production Systems (AR5, WG3, Chapter 7), and Human Health: Impacts, Adaptation, and Co-Benefits (AR5, WG2, Chapter 11). Many of the aspects discussed in the article are reviewed at great length there, and in part using more recent studies. I would at least expect that a review like the present one would mention those reports, so that interested individuals know where they can find more detailed information.

 The section on stratospheric ozone depletion is a good case in point. The impacts of changes in ultraviolet radiation on biomass are reviewed, but it is not clear whether it is an important effect or not. For example, what is missing from the discussion is the fact that the ozone hole has started to “heal” (see, e.g., Solomon et al, Science 2016, ^1^ ; or an earlier IPCC special report on the ozone layer), and where to read on. In addition to the agricultural impacts, changes in ultraviolet radiation also impact human health directly. It might be worth re-emphasizing that the direct health impacts of many of the environmental changes reviewed are not discussed in the article. (That is also the case for tropospheric ozone, which is briefly mentioned in relation to oxidative stress for plants, but which arguably has a bigger direct health impact in its relationship to urban air pollution).

 At a couple of instances, it might be worth to add some detail related to attribution. For example, in the discussion on acid rain (3.5), one could get away with the impression that it is a natural phenomenon (“Acid rains generally result from the reaction of water molecules and sulphur dioxide or nitrogen oxide in the atmosphere,” p. 6). Whilst natural phenomena, such as volcanic eruptions, surely contribute to acid-rain precursors, the principal causes are anthropogenic emissions of sulphur and nitrogen compounds, especially from coal-fired power plants. Another clarification regarding attribution might be when discussing fruit and vegetable consumption. On page 8, it is mentioned that in some situation, low consumption reflects population preferences. Although one can surely see it that way, another way of explaining consumption behaviour is by pointing to the food environment and its role in shaping preferences. The benefit of this angle is that it allows one to study the influences of actors, such as governments and the food industry, on the food environment and on the preferences shaped by it.

 Despite being in the title, health is actually not discussed to a great extent in the review. That’s totally fine, but it might be worth being a bit clearer about what is, and what is not discussed in the article. A specific aspect I was missing from the discussion of pathways leading from nutrition to population health (pp. 7-8) is dietary composition. What is mentioned are the quantity and quality of food intake. Although dietary composition is sometimes subsumed under the banner of quality of food intake, that is not obvious from the related paragraph and could be clarified. Of note here is that changes in dietary composition are broader, and more impactful for health than changes in specific nutrient levels – a point illustrated by the ranking of risk factors in the Global Burden of Disease study ^2^ that is referred to a couple of times in the article.  

 A final comment is that the literature used could be a bit more general at times. For example, I don’t understand why when discussing the greenhouse gas emissions related to agriculture, the only study referred to for quantifying the emissions attributable to fruit and vegetable consumption is a working paper focussed on the UK. There are several more general sources that have quantified the emissions attributable to both global and regional consumption of fruits and vegetables. For example, in one of my own studies ^3^, I calculated that about 7% of all food-related greenhouse gas emissions in 2005/07 were related to fruit and vegetable consumption. Tilman and Clark’s article ^4^ also includes some global estimates and could be consulted in that regard. Another example is the discussion on changes in water demand (p. 6) where a national case-study on India is cited, without noting more comprehensive, global analyses. Good resources here are again the IPCC, and the Agricultural Model Intercomparison and Improvement Project (AgMIP). In general, I think it is good practice in reviews to indicate whether a reference provides a specific example, or whether it supports a general argument.     

 Good luck with the revisions. I enjoyed reading the article.

I have read this submission. I believe that I have an appropriate level of expertise to confirm that it is of an acceptable scientific standard, however I have significant reservations, as outlined above.

## References

[1] SolomonSIvyDJKinnisonDMillsMJNeelyRRSchmidtA: Emergence of healing in the Antarctic ozone layer. *Science*.2016;353(6296) : 10.1126/science.aae0061 269-74 10.1126/science.aae0061 27365314

[2] GBD 2015 Risk Factors Collaborators: Global, regional, and national comparative risk assessment of 79 behavioural, environmental and occupational, and metabolic risks or clusters of risks, 1990-2015: a systematic analysis for the Global Burden of Disease Study 2015. *Lancet*.2016;388(10053) : 10.1016/S0140-6736(16)31679-8 1659-1724 10.1016/S0140-6736(16)31679-8 PMC538885627733284

[3] SpringmannMGodfrayHCRaynerMScarboroughP: Analysis and valuation of the health and climate change cobenefits of dietary change. *Proc Natl Acad Sci U S A*.2016;113(15) : 10.1073/pnas.1523119113 4146-51 10.1073/pnas.1523119113 27001851PMC4839446

[4] TilmanDClarkM: Global diets link environmental sustainability and human health. *Nature*.2014;515(7528) : 10.1038/nature13959 518-22 10.1038/nature13959 25383533

# Response

**Published:** 2017-10-17

**Authors:** Hanna Tuomisto

**Affiliations:** London School of Hygiene & Tropical Medicine, UK

**Responses to Reviewer 2**

*Dear Dr Springmann,*

*Thank you very much for your excellent comments that have helped us to improve our paper. We have revised the paper based on your suggestions as detailed below. In addition, we made revisions based on comments from Dr McDermott and we added a new section discussing adaptation and mitigation options (section 7).*

 “In their article ‘Effects of environmental change on population nutrition and health: A comprehensive framework with a focus on fruits and vegetables,’ Hanna Tuomisto and colleagues aim to develop a framework that details the interactions between environmental change, diets, and health, with a particular focus on fruits and vegetables. Their article is a welcome review of the impacts of environmental change on agriculture and health, and I recommend it for indexing subject to addressing a few comments that I am detailing below.

 First, I think the motivation of the review could be strengthened. What sets it apart from other reviews, such as the IPCC’s, or maps of the food system?”

*Authors: We appreciate the motivation/difference was not stipulated clearly: the focus on fruits and vegetables rather than on staple crops. We have strengthened the justification for this in the introduction section.*

 “Related to that, the article does not contain any methods and discussion sections. This might be fine for a review/overview article, but if the stated aim is to develop a framework of interactions, then one would expect at least some detail on what the added value of that framework is, how it was constructed, and how it compares to other frameworks.”

*Authors: We have expanded the description of the methods, and added a methods heading. We have also strengthened the explanation of the added value and differences compared to the other frameworks. A brief discussion of the potential uses of the framework as well as some limitations can be found in section 8.*

 “From my reading of the article, it is a review of interactions between environmental change and mostly agriculture, with special emphasis on the implications for fruits and vegetables, and some discussion on health implications. It might therefore be advisable to describe it as such.”

*Authors: The title and introduction of the paper have been amended to clarify that it presents a framework with a particular emphasis on fruit and vegetable production. As the paper is designed to be read by a primarily health-focused audience, we have added particular detail on the interactions between environmental change and fruit and vegetable production, as this is the area of the framework the journal’s readership is likely to be least familiar with.*

 “That would also address some problems I have with the conclusions, which seem to be a little bit of an overstretch to me. For most of the points raised, what would actually be required is some information on the relative importance of each factor. For advocacy or funding purposes, for example, one would want to know how significant a particular aspect is to gauge whether focussing on it would be worth the investment. The review, I think, nicely catalogues the various interactions between environmental change and agriculture, but it does not contain any interpretation of the information that is presented, or a discussion on what to do with it.”

*Authors: The aim of the framework is to provide a basis for modelling and quantification of the relative importance of the different factors, and as such the quantification itself is beyond the scope of this piece of work. However, we also identified some uses for the framework itself, which are listed in the conclusions section. We will look into possibilities for other research groups to add to the framework in the future (perhaps using open source software) to further quantify each of the indicated links. * 

 “For some of the aspects that are discussed I found myself going back to related IPCC chapters, in particular those on Agriculture, Forestry and Other Land Use (AR5, WG3, Chapter 11), Food Security and Food Production Systems (AR5, WG3, Chapter 7), and Human Health: Impacts, Adaptation, and Co-Benefits (AR5, WG2, Chapter 11). Many of the aspects discussed in the article are reviewed at great length there, and in part using more recent studies. I would at least expect that a review like the present one would mention those reports, so that interested individuals know where they can find more detailed information.”

*Authors: Thank you for the suggestion. We have added citations to the suggested reports in the paper.*

 “The section on stratospheric ozone depletion is a good case in point. The impacts of changes in ultraviolet radiation on biomass are reviewed, but it is not clear whether it is an important effect or not. For example, what is missing from the discussion is the fact that the ozone hole has started to “heal” (see, e.g., Solomon et al, Science 2016, ^1^ ; or an earlier IPCC special report on the ozone layer), and where to read on.”

*Authors: Thank you for the comment. We have added the point that the ozone layer is healing and added a reference to the Solomon et al 2016 paper. We also removed the following sentence as the reference is relatively old and is contradicting the fact that the ozone layer is healing: “It has been estimated that the springtime UV doses will increase 14% in the Northern hemisphere and 40% in the Southern hemisphere in 2010–2020 compared to levels in 1979–1992 (Taalas et al., 2000).”.*

 “In addition to the agricultural impacts, changes in ultraviolet radiation also impact human health directly. It might be worth re-emphasizing that the direct health impacts of many of the environmental changes reviewed are not discussed in the article. (That is also the case for tropospheric ozone, which is briefly mentioned in relation to oxidative stress for plants, but which arguably has a bigger direct health impact in its relationship to urban air pollution).”

*Authors: We have added a note in the introduction section (end of the fourth paragraph) stating the fact that the paper doesn’t cover direct health impacts.*

 “At a couple of instances, it might be worth to add some detail related to attribution. For example, in the discussion on acid rain (3.5), one could get away with the impression that it is a natural phenomenon (“Acid rains generally result from the reaction of water molecules and sulphur dioxide or nitrogen oxide in the atmosphere,” p. 6). Whilst natural phenomena, such as volcanic eruptions, surely contribute to acid-rain precursors, the principal causes are anthropogenic emissions of sulphur and nitrogen compounds, especially from coal-fired power plants.”

*Authors: We have clarified the point on acid rains and screened the paper for additional paragraphs that would benefit from more detail related to attribution: more detail was added to these sections.*

 “Another clarification regarding attribution might be when discussing fruit and vegetable consumption. On page 8, it is mentioned that in some situation, low consumption reflects population preferences. Although one can surely see it that way, another way of explaining consumption behaviour is by pointing to the food environment and its role in shaping preferences. The benefit of this angle is that it allows one to study the influences of actors, such as governments and the food industry, on the food environment and on the preferences shaped by it.”

*Authors: Thank you for this excellent comment. We edited the sentence to: “A remarkable example of these feedback loops is based on the consumer-driven rapid global shift towards a more “Western” diet, which is driven by urbanisation, economic growth and changes in technology and culture ( Popkin, 2006).”*

 “Despite being in the title, health is actually not discussed to a great extent in the review. That’s totally fine, but it might be worth being a bit clearer about what is, and what is not discussed in the article.”

*Authors: we have now clarified the desired focus of the paper, expanded the health section (5.2) and briefly discussed possible implications for health.*

 “A specific aspect I was missing from the discussion of pathways leading from nutrition to population health (pp. 7-8) is dietary composition. What is mentioned are the quantity and quality of food intake. Although dietary composition is sometimes subsumed under the banner of quality of food intake, that is not obvious from the related paragraph and could be clarified. Of note here is that changes in dietary composition are broader, and more impactful for health than changes in specific nutrient levels – a point illustrated by the ranking of risk factors in the Global Burden of Disease study ^2^ that is referred to a couple of times in the article.”  

*Authors: we clarified that the term ‘food quality’ covers also dietary composition, and have altered this section to focus more explicitly on fruits and vegetables and their contribution to quality of dietary intake.*

 “A final comment is that the literature used could be a bit more general at times. For example, I don’t understand why when discussing the greenhouse gas emissions related to agriculture, the only study referred to for quantifying the emissions attributable to fruit and vegetable consumption is a working paper focussed on the UK. There are several more general sources that have quantified the emissions attributable to both global and regional consumption of fruits and vegetables. For example, in one of my own studies ^3^, I calculated that about 7% of all food-related greenhouse gas emissions in 2005/07 were related to fruit and vegetable consumption.”

*Authors: thanks for this information. We have added a reference to your paper.*

 “Tilman and Clark’s article ^4^ also includes some global estimates and could be consulted in that regard. Another example is the discussion on changes in water demand (p. 6) where a national case-study on India is cited, without noting more comprehensive, global analyses. Good resources here are again the IPCC, and the Agricultural Model Intercomparison and Improvement Project (AgMIP). In general, I think it is good practice in reviews to indicate whether a reference provides a specific example, or whether it supports a general argument.”     

*Authors: We have improved this section and added a reference to the IPCC report.*

**References**

 1. Solomon S, Ivy DJ, Kinnison D, Mills MJ, Neely RR, Schmidt A: Emergence of healing in the Antarctic ozone layer. *Science*. 2016; **353** (6296): 269-74 PubMed Abstract | Publisher Full Text 

 2. GBD 2015 Risk Factors Collaborators: Global, regional, and national comparative risk assessment of 79 behavioural, environmental and occupational, and metabolic risks or clusters of risks, 1990-2015: a systematic analysis for the Global Burden of Disease Study 2015. *Lancet*. 2016; **388** (10053): 1659-1724 Publisher Full Text 

 3. Springmann M, Godfray HC, Rayner M, Scarborough P: Analysis and valuation of the health and climate change cobenefits of dietary change. *Proc Natl Acad Sci U S A*. 2016; **113** (15): 4146-51 PubMed Abstract | Publisher Full Text 

 4. Tilman D, Clark M: Global diets link environmental sustainability and human health. *Nature*. 2014; **515** (7528): 518-22 PubMed Abstract | Publisher Full Text

# Peer review: Referee response for version 1

**DOI:** 10.21956/wellcomeopenres.12073.r22290

**Published:** 2017-04-27

**Authors:** John McDermott

**Affiliations:** 1 CGIAR Research Program on Agriculture for Nutrition and Health (A4NH), International Food Policy Research Institute (IFPRI), Washington, DC, USA

The objective of this paper is to provide a comprehensive framework for the effects of environmental change on population nutrition and health.  The authors correctly, in my opinion, advocate for a more comprehensive approach that considers multiple disciplines and key interactions between the environment, food production and population nutrition and health. The framework is less comprehensive than the title, restricting itself to environmental change and a subset of food production from major crops, and extending that to fruits and vegetables. These could be brought into alignment with revisions by rephrasing the title and narrowing the scope to focus on the subset of issues addressed. If a more comprehensive approach, addressing issues raised below, is desired, the paper would need to be changed much more dramatically.

 My comments focus on the utility of the framework for policies and actions for linking the environment, food production, and population nutrition and health in low- and middle-income countries (LMICs). In general, the framework proposed has important elements but seems better suited to the context of high-income countries.  Agriculture is the sector with the greatest influence on natural systems globally, and it is changing rapidly in LMICs. Some of the biggest environmental influences of agriculture on the environment in LMICs are:

1. expansion of agricultural lands into natural forests,
2. intensification of livestock and fish systems (that can have beneficial or negative effects, depending on management),
3. depletion of ground water, and
4. land / soil degradation.

All these agricultural changes have important implications for greenhouse gas production and climate change adaptation and mitigation. For all these topics, there are important interactions between agriculture and the environment which have implications for population nutrition and health. As issues 1-3 are not considered in the paper, the comprehensive framework proposed does not adequately address some of the biggest food system issues in LMICs.

 In particular, for a paper linking environmental change to population nutrition and health through food, the failure to consider animal production (livestock and fish) is a profound omission. In smallholder systems across Africa and Asia, mixed farming with both animals and plants is very common. The combination of plants and animal production are synergistic – socio-economically and biologically.

 The methodology followed has led to a useful initial framework. However, if this framework is to be more generally applicable in LMICs (as is implied) I would suggest that the current framework be revised considering the general points above and some additional, more specific, points below. These points highlight some of the many tradeoffs and challenges that decision makers struggle with in the environment, food, and health nexus that a comprehensive framework needs to consider.

  

1. Water: water quality is an important issue and is intimately linked with food safety, particularly for vegetables. The landmark WHO Foodborne Disease Burden Epidemiology Reference Group (FERG) report from December 2015 estimates that the main burdens associated with fresh foods are overwhelmingly due to biological pathogens rather than chemicals ( http://www.who.int/foodsafety/areas_work/foodborne-diseases/ferg/en/). In general, consumers are more concerned with chemical contamination. Many of the vegetables consumed in urban and peri-urban areas are grown with contaminated wastewater. How this wastewater is managed is a critical issue for vegetable production ^1^. The issue of water availability is ignored in the framework, but it is of critical importance. Subsidized water for cereal production is leading to a depletion of groundwater in the western Indo-Gangetic plains. Over-exploitation of ground water is also critical in dryland farming areas in Australia, Central Asia and North America. These systems will be forced to de-intensify or become unproductive. In Africa, there has been relatively little investment in irrigation to date, but it is a very dry continent and sustainable irrigation will be critical to adapting food production systems to increasing climate variability.
2. Biodiversity loss: this is one example of the need to go beyond listing issues to assessing tradeoffs. As noted by the authors, this is complex to model and decide, but people are constantly making decisions between adhering to a precautionary principle of maintaining natural areas, and adopting more intensive and less diverse systems. The framework would need to consider how such tradeoffs can be considered and monitored, and evolve over time.
3. Diet quality in sustainable and healthy food systems: implied in the discussion of fruits and vegetables is the diversification of diets and improving diet quality by promoting consumption of healthy foods (and reducing consumption of unhealthy foods). In LMICs, most agricultural policies provide subsidies and greater investment for cereals with the result that supply chains for cereals are more efficient and the prices lower relative to more nutritious foods such as pulses, fish and vegetables. Thus, rebalancing agricultural policies to make them more commodity-neutral is needed to improve diet quality.
4. Tradeoffs between sustainability and health. Animal-source foods represent the greatest challenge in this regard since they are very nutritious but much more environmentally costly. A strong case can be made that the poor (especially mothers and children) should eat more animal-source foods, but it is desirable, for both sustainability and health reasons, to limit the dramatic increases in consumption of animal-source foods observed as incomes rise in LMICs.                        

In revising the framework, these are some key issues to consider. It might be useful to get other inputs to adapt or add that a functional comprehensive framework should address.

I have read this submission. I believe that I have an appropriate level of expertise to confirm that it is of an acceptable scientific standard, however I have significant reservations, as outlined above.

## References

[1] Antwi-AgyeiPCairncrossSPeaseyAPriceVBruceJBakerKMoeCAmpofoJArmahGEnsinkJ: A Farm to Fork Risk Assessment for the Use of Wastewater in Agriculture in Accra, Ghana. *PLoS One*.2015;10(11) : 10.1371/journal.pone.0142346 e0142346 10.1371/journal.pone.0142346 26556044PMC4640866

# Response

**Published:** 2017-10-03

**Authors:** Hanna Tuomisto

**Affiliations:** London School of Hygiene & Tropical Medicine, UK

**Responses to Reviewer 1**

*Dear Dr McDermott,*

*Thank you very much for your excellent comments that have helped us to improve our paper. We have revised the paper based on your suggestions as detailed below. In addition, we made revisions based on comments from Dr Springmann and we added a new section discussing adaptation and mitigation options (section 7).*

“The objective of this paper is to provide a comprehensive framework for the effects of environmental change on population nutrition and health.  The authors correctly, in my opinion, advocate for a more comprehensive approach that considers multiple disciplines and key interactions between the environment, food production and population nutrition and health. The framework is less comprehensive than the title, restricting itself to environmental change and a subset of food production from major crops, and extending that to fruits and vegetables.”

*Authors: we have changed the title, so that it reflects the focus of the paper better (fruits and vegetables). The new title is: Effects of environmental change on agriculture, population nutrition and health: A framework with a focus on fruits and vegetables*

“These could be brought into alignment with revisions by rephrasing the title and narrowing the scope to focus on the subset of issues addressed. If a more comprehensive approach, addressing issues raised below, is desired, the paper would need to be changed much more dramatically.”

*Authors: we have revised the paper throughout to be clearly focused on fruits and vegetables.*

“My comments focus on the utility of the framework for policies and actions for linking the environment, food production, and population nutrition and health in low- and middle-income countries (LMICs). In general, the framework proposed has important elements but seems better suited to the context of high-income countries.  Agriculture is the sector with the greatest influence on natural systems globally, and it is changing rapidly in LMICs. Some of the biggest environmental influences of agriculture on the environment in LMICs are:

1. expansion of agricultural lands into natural forests,
2. intensification of livestock and fish systems (that can have beneficial or negative effects, depending on management),
3. depletion of ground water, and
4. land / soil degradation.

All these agricultural changes have important implications for greenhouse gas production and climate change adaptation and mitigation. For all these topics, there are important interactions between agriculture and the environment which have implications for population nutrition and health. As issues 1-3 are not considered in the paper, the comprehensive framework proposed does not adequately address some of the biggest food system issues in LMICs.”

*Authors: we agree with the reviewer about the many interactions between agriculture and the environment. It would be very useful to further explore all of these in detail, and this is certainly something we would like to commit to in our future research. For this first paper, we decided to describe the impacts of environmental changes on agriculture and have now further clarified in the text that this was our focus. In a future paper, we could subsequently look at the impacts of agriculture on the environment: for now these are only briefly discussed in section 6. Depletion of groundwater is briefly covered in section 4.4, which we have slightly expanded. We appreciate the reviewer’s comments concerning the relevance of the framework to LMICs as well as high income countries: we have now made sure more LMIC examples have been added throughout the revised manuscript.*

“In particular, for a paper linking environmental change to population nutrition and health through food, the failure to consider animal production (livestock and fish) is a profound omission. In smallholder systems across Africa and Asia, mixed farming with both animals and plants is very common. The combination of plants and animal production are synergistic – socio-economically and biologically.”

*Authors: we agree with this comment and have improved the reasoning why the paper focuses on fruits and vegetables in the introduction section. * 

“The methodology followed has led to a useful initial framework. However, if this framework is to be more generally applicable in LMICs (as is implied) I would suggest that the current framework be revised considering the general points above and some additional, more specific, points below. These points highlight some of the many tradeoffs and challenges that decision makers struggle with in the environment, food, and health nexus that a comprehensive framework needs to consider.

 

1. Water: water quality is an important issue and is intimately linked with food safety, particularly for vegetables. The landmark WHO Foodborne Disease Burden Epidemiology Reference Group (FERG) report from December 2015 estimates that the main burdens associated with fresh foods are overwhelmingly due to biological pathogens rather than chemicals (http://www.who.int/foodsafety/areas_work/foodborne-diseases/ferg/en/). In general, consumers are more concerned with chemical contamination. Many of the vegetables consumed in urban and peri-urban areas are grown with contaminated wastewater. How this wastewater is managed is a critical issue for vegetable production (for example, see https://www.ncbi.nlm.nih.gov/pmc/articles/PMC4640866/).”

*Authors: thank you for this comment. We have added the issue of pathogen contaminated irrigation water with the above references in section 4.3.*

“The issue of water availability is ignored in the framework, but it is of critical importance. Subsidized water for cereal production is leading to a depletion of groundwater in the western Indo-Gangetic plains. Over-exploitation of ground water is also critical in dryland farming areas in Australia, Central Asia and North America. These systems will be forced to de-intensify or become unproductive. In Africa, there has been relatively little investment in irrigation to date, but it is a very dry continent and sustainable irrigation will be critical to adapting food production systems to increasing climate variability.”

*Authors: We appreciate that the issue of water availability was not appropriately covered and was only partly included in the “non-renewable resource depletion” section of the framework (section 4.4). We have now expanded the text explaining the issue of water availability and referencing the points made by the reviewer above. *

1. “Biodiversity loss: this is one example of the need to go beyond listing issues to assessing tradeoffs. As noted by the authors, this is complex to model and decide, but people are constantly making decisions between adhering to a precautionary principle of maintaining natural areas, and adopting more intensive and less diverse systems. The framework would need to consider how such tradeoffs can be considered and monitored, and evolve over time.”

*Authors: we agree that trade-offs are very important to consider, but feel that exploring them is beyond the scope of a structural framework. Trade-offs between the links and interactions identified here will be addressed in the future modelling work that will be carried out by using the framework.*

“Diet quality in sustainable and healthy food systems: implied in the discussion of fruits and vegetables is the diversification of diets and improving diet quality by promoting consumption of healthy foods (and reducing consumption of unhealthy foods). In LMICs, most agricultural policies provide subsidies and greater investment for cereals with the result that supply chains for cereals are more efficient and the prices lower relative to more nutritious foods such as pulses, fish and vegetables. Thus, rebalancing agricultural policies to make them more commodity-neutral is needed to improve diet quality.”

*Authors: thank you for the helpful comment. This is again an issue that we can explore in our future modelling work, and is mentioned in section 8.*

1. “Tradeoffs between sustainability and health. Animal-source foods represent the greatest challenge in this regard since they are very nutritious but much more environmentally costly. A strong case can be made that the poor (especially mothers and children) should eat more animal-source foods, but it is desirable, for both sustainability and health reasons, to limit the dramatic increases in consumption of animal-source foods observed as incomes rise in LMICs.”

*Authors: as we have now narrowed the focus of the framework following reviewer suggestions to focus more explicitly on fruits and vegetables, animal-source foods are beyond the scope of the paper. We agree that it will be extremely important to explore trade-offs between sustainability and health in our future modelling work, however.*

                        

**Responses to Reviewer 2**

*Dear Dr Springmann,*

*Thank you very much for your excellent comments that have helped us to improve our paper. We have revised the paper based on your suggestions as detailed below. In addition, we made revisions based on comments from Dr McDermott and we added a new section discussing adaptation and mitigation options (section 7).*

“In their article ‘Effects of environmental change on population nutrition and health: A comprehensive framework with a focus on fruits and vegetables,’ Hanna Tuomisto and colleagues aim to develop a framework that details the interactions between environmental change, diets, and health, with a particular focus on fruits and vegetables. Their article is a welcome review of the impacts of environmental change on agriculture and health, and I recommend it for indexing subject to addressing a few comments that I am detailing below.

First, I think the motivation of the review could be strengthened. What sets it apart from other reviews, such as the IPCC’s, or maps of the food system?”

*Authors: We appreciate the motivation/difference was not stipulated clearly: the focus on fruits and vegetables rather than on staple crops. We have strengthened the justification for this in the introduction section.*

“Related to that, the article does not contain any methods and discussion sections. This might be fine for a review/overview article, but if the stated aim is to develop a framework of interactions, then one would expect at least some detail on what the added value of that framework is, how it was constructed, and how it compares to other frameworks.”

*Authors: We have expanded the description of the methods, and added a methods heading. We have also strengthened the explanation of the added value and differences compared to the other frameworks. A brief discussion of the potential uses of the framework as well as some limitations can be found in section 8.*

“From my reading of the article, it is a review of interactions between environmental change and mostly agriculture, with special emphasis on the implications for fruits and vegetables, and some discussion on health implications. It might therefore be advisable to describe it as such.”

*Authors: The title and introduction of the paper have been amended to clarify that it presents a framework with a particular emphasis on fruit and vegetable production. As the paper is designed to be read by a primarily health-focused audience, we have added particular detail on the interactions between environmental change and fruit and vegetable production, as this is the area of the framework the journal’s readership is likely to be least familiar with.*

“That would also address some problems I have with the conclusions, which seem to be a little bit of an overstretch to me. For most of the points raised, what would actually be required is some information on the relative importance of each factor. For advocacy or funding purposes, for example, one would want to know how significant a particular aspect is to gauge whether focussing on it would be worth the investment. The review, I think, nicely catalogues the various interactions between environmental change and agriculture, but it does not contain any interpretation of the information that is presented, or a discussion on what to do with it.”

*Authors: The aim of the framework is to provide a basis for modelling and quantification of the relative importance of the different factors, and as such the quantification itself is beyond the scope of this piece of work. However, we also identified some uses for the framework itself, which are listed in the conclusions section. We will look into possibilities for other research groups to add to the framework in the future (perhaps using open source software) to further quantify each of the indicated links. * 

“For some of the aspects that are discussed I found myself going back to related IPCC chapters, in particular those on Agriculture, Forestry and Other Land Use (AR5, WG3, Chapter 11), Food Security and Food Production Systems (AR5, WG3, Chapter 7), and Human Health: Impacts, Adaptation, and Co-Benefits (AR5, WG2, Chapter 11). Many of the aspects discussed in the article are reviewed at great length there, and in part using more recent studies. I would at least expect that a review like the present one would mention those reports, so that interested individuals know where they can find more detailed information.”

*Authors: Thank you for the suggestion. We have added citations to the suggested reports in the paper.*

“The section on stratospheric ozone depletion is a good case in point. The impacts of changes in ultraviolet radiation on biomass are reviewed, but it is not clear whether it is an important effect or not. For example, what is missing from the discussion is the fact that the ozone hole has started to “heal” (see, e.g., Solomon et al, Science 2016, ^1^ ; or an earlier IPCC special report on the ozone layer), and where to read on.”

*Authors: Thank you for the comment. We have added the point that the ozone layer is healing and added a reference to the Solomon et al 2016 paper. We also removed the following sentence as the reference is relatively old and is contradicting the fact that the ozone layer is healing: “It has been estimated that the springtime UV doses will increase 14% in the Northern hemisphere and 40% in the Southern hemisphere in 2010–2020 compared to levels in 1979–1992 (Taalas et al., 2000).”.*

“In addition to the agricultural impacts, changes in ultraviolet radiation also impact human health directly. It might be worth re-emphasizing that the direct health impacts of many of the environmental changes reviewed are not discussed in the article. (That is also the case for tropospheric ozone, which is briefly mentioned in relation to oxidative stress for plants, but which arguably has a bigger direct health impact in its relationship to urban air pollution).”

*Authors: We have added a note in the introduction section (end of the fourth paragraph) stating the fact that the paper doesn’t cover direct health impacts.*

“At a couple of instances, it might be worth to add some detail related to attribution. For example, in the discussion on acid rain (3.5), one could get away with the impression that it is a natural phenomenon (“Acid rains generally result from the reaction of water molecules and sulphur dioxide or nitrogen oxide in the atmosphere,” p. 6). Whilst natural phenomena, such as volcanic eruptions, surely contribute to acid-rain precursors, the principal causes are anthropogenic emissions of sulphur and nitrogen compounds, especially from coal-fired power plants.”

*Authors: We have clarified the point on acid rains and screened the paper for additional paragraphs that would benefit from more detail related to attribution: more detail was added to these sections.*

“Another clarification regarding attribution might be when discussing fruit and vegetable consumption. On page 8, it is mentioned that in some situation, low consumption reflects population preferences. Although one can surely see it that way, another way of explaining consumption behaviour is by pointing to the food environment and its role in shaping preferences. The benefit of this angle is that it allows one to study the influences of actors, such as governments and the food industry, on the food environment and on the preferences shaped by it.”

*Authors: Thank you for this excellent comment. We edited the sentence to: “A remarkable example of these feedback loops is based on the consumer-driven rapid global shift towards a more “Western” diet, which is driven by urbanisation, economic growth and changes in technology and culture ( Popkin, 2006).”*

“Despite being in the title, health is actually not discussed to a great extent in the review. That’s totally fine, but it might be worth being a bit clearer about what is, and what is not discussed in the article.”

*Authors: we have now clarified the desired focus of the paper, expanded the health section (5.2) and briefly discussed possible implications for health.*

“A specific aspect I was missing from the discussion of pathways leading from nutrition to population health (pp. 7-8) is dietary composition. What is mentioned are the quantity and quality of food intake. Although dietary composition is sometimes subsumed under the banner of quality of food intake, that is not obvious from the related paragraph and could be clarified. Of note here is that changes in dietary composition are broader, and more impactful for health than changes in specific nutrient levels – a point illustrated by the ranking of risk factors in the Global Burden of Disease study ^2^ that is referred to a couple of times in the article.”  

*Authors: we clarified that the term ‘food quality’ covers also dietary composition, and have altered this section to focus more explicitly on fruits and vegetables and their contribution to quality of dietary intake.*

“A final comment is that the literature used could be a bit more general at times. For example, I don’t understand why when discussing the greenhouse gas emissions related to agriculture, the only study referred to for quantifying the emissions attributable to fruit and vegetable consumption is a working paper focussed on the UK. There are several more general sources that have quantified the emissions attributable to both global and regional consumption of fruits and vegetables. For example, in one of my own studies ^3^, I calculated that about 7% of all food-related greenhouse gas emissions in 2005/07 were related to fruit and vegetable consumption.”

*Authors: thanks for this information. We have added a reference to your paper.*

“Tilman and Clark’s article ^4^ also includes some global estimates and could be consulted in that regard. Another example is the discussion on changes in water demand (p. 6) where a national case-study on India is cited, without noting more comprehensive, global analyses. Good resources here are again the IPCC, and the Agricultural Model Intercomparison and Improvement Project (AgMIP). In general, I think it is good practice in reviews to indicate whether a reference provides a specific example, or whether it supports a general argument.”     

*Authors: We have improved this section and added a reference to the IPCC report.*

**References**

1. Solomon S, Ivy DJ, Kinnison D, Mills MJ, Neely RR, Schmidt A: Emergence of healing in the Antarctic ozone layer. *Science*. 2016; **353** (6296): 269-74 PubMed Abstract | Publisher Full Text 

2. GBD 2015 Risk Factors Collaborators: Global, regional, and national comparative risk assessment of 79 behavioural, environmental and occupational, and metabolic risks or clusters of risks, 1990-2015: a systematic analysis for the Global Burden of Disease Study 2015. *Lancet*. 2016; **388** (10053): 1659-1724 Publisher Full Text 

3. Springmann M, Godfray HC, Rayner M, Scarborough P: Analysis and valuation of the health and climate change cobenefits of dietary change. *Proc Natl Acad Sci U S A*. 2016; **113** (15): 4146-51 PubMed Abstract | Publisher Full Text 

4. Tilman D, Clark M: Global diets link environmental sustainability and human health. *Nature*. 2014; **515** (7528): 518-22 PubMed Abstract | Publisher Full Text
